# Leptomeningeal Contrast Enhancement Is Associated with Disability Progression and Grey Matter Atrophy in Multiple Sclerosis

**DOI:** 10.1155/2017/8652463

**Published:** 2017-10-02

**Authors:** Gleb Makshakov, Evgeniy Magonov, Natalia Totolyan, Vladimir Nazarov, Sergey Lapin, Alexandra Mazing, Elena Verbitskaya, Tatiana Trofimova, Vladimir Krasnov, Maria Shumilina, Alexander Skoromets, Evgeniy Evdoshenko

**Affiliations:** ^1^SBIH City Clinical Hospital No. 31, City Center of MS and Autoimmune Diseases, St. Petersburg, Russia; ^2^Neurology Department, FSBEI HE I.P. Pavlov SPbSMU MOH Russia, St. Petersburg, Russia; ^3^Institute of Human Brain of the Russian Academy of Sciences, St. Petersburg, Russia; ^4^Center for Molecular Medicine, Laboratory of Autoimmune Diagnostics, FSBEI HE I.P. Pavlov SPbSMU MOH Russia, St. Petersburg, Russia; ^5^Department of Clinical Pharmacology and EBM, FSBEI HE I.P. Pavlov SPbSMU MOH Russia, St. Petersburg, Russia; ^6^Department of Pharmacoepidemiology and Biostatistics, FSBEI HE I.P. Pavlov SPbSMU MOH Russia, St. Petersburg, Russia; ^7^Institute of Experimental Medicine, St. Petersburg, Russia

## Abstract

Leptomeningeal contrast enhancement (LMCE) on magnetic resonance imaging (MRI) is a newly recognized possible biomarker in multiple sclerosis (MS), associated with MS progression and cortical atrophy. In this study, we aimed to assess the prevalence of LMCE foci and their impact on neurodegeneration and disability.* Materials.* 54 patients with MS were included in the study. LMCE were detected with a 3 Tesla scanner on postcontrast fluid-attenuated inversion-recovery (FLAIR) sequence. Expanded Disability Status Scale (EDSS) score, number of relapses during 5 years from MS onset, and number of contrast-enhancing lesions on T1 weighted MRI were counted.* Results.* LMCE was detected in 41% (22/54) of patients. LMCE-positive patients had longer disease duration (*p* = 0,0098) and higher EDSS score (*p* = 0,039), but not a higher relapse rate (*p* = 0,091). No association of LMCE with higher frequency of contrast-enhancing lesions on T1-weighted images was detected (*p* = 0,3842). Analysis of covariates, adjusted for age, sex, and disease duration, revealed a significant effect of LMCE on the cortex volume (*p* = 0.043, *F* = 2.529), the total grey matter volume (*p* = 0.043, *F* = 2.54), and total ventricular volume (*p* = 0.039, *F* = 2.605).* Conclusions.* LMCE was shown to be an independent and significant biomarker of grey matter atrophy and disability in MS.

## 1. Introduction

Multiple sclerosis is a chronic debilitating disease of the central nervous system (CNS). Recent studies have demonstrated a prominent neurodegenerative component starts early in the disease course [1, 2]. Grey matter lesions, detected with double inversion-recovery (DIR) sequence, and grey matter atrophy have been shown to contribute to the pathogenesis of neurodegeneration and have stronger correlations with disability than white matter lesions and total brain atrophy [3, 4]. Grey matter pathology in MS is prominent in both deep grey matter structures (thalamus, etc.) and cortex. Cortical pathology has been shown to be prominent in advanced stages of secondary and primary progressive MS [5]. A relation between leptomeningeal ectopic lymphocytic aggregates and cortical pathology was first shown in the study of Serafini et al. [6]. In the study by Magliozzi et al., a gradient of necroptosis and demyelination severity in the cortex under these leptomeningeal aggregates with maximal intensity in subpial areas has been demonstrated [7]. In another study, these aggregates were localized widely across the brain in subarachnoid space, lying in depths of cortical sulci [8]. Studies with immunostaining have demonstrated massive B-cell infiltration to be a part of such aggregates [6, 8]. These B-cell enriched aggregates are probably related to the intrathecal oligoclonal bands (OCB) of IgG and/or IgM and Ig-free light chains (FLC) production as well as an increased level of specific antiviral antibodies (positive MRZ-reaction).

Detection of leptomeningeal pathology in MS is a difficult task due to a small size of leptomeningeal infiltrates which are usually less than 1 mm [9]. Manifest accumulation of contrast agent on T1 postcontrast MRI due to ubiquitous vascularization of meninges makes it difficult to distinguish foci of meningeal pathology from the normal tissue. Fluid-attenuated inversion-recovery (FLAIR) has been shown to detect smaller concentrations of gadolinium (Gd) contrast agent than T1 sequence [10]. Possibility of postcontrast FLAIR sequence to detect foci of leptomeningeal enhancement in MS with long disease duration has been shown recently. In a study by Absinta et al. two autopsy samples were compared with prior FLAIR postcontrast images, and the foci of leptomeningeal contrast enhancement were shown to be correlated with the meningeal infiltrates and underlying cortical demyelination [11]. Studies on the prevalence of leptomeningeal contrast enhancement (LMCE) have demonstrated contradictory results. The study by Eisele et al. has demonstrated a low sensitivity of the proposed MRI protocol for the detection of LMCE in early course of MS [12], while in the study by Absinta et al. the prevalence of LMCE was shown to be 25% of cases [11]. In the prospective study by Zivadinov et al. a relationship between LMCE and progressive grey matter pathology was shown [9]. Therefore LMCE is, presumably, a biomarker of disease severity and more data are required to determine prognostic significance of LMCE on brain atrophy and disability in MS. Also, studies are required to detect its relationship with the biomarkers of B-cell infiltration and activation.

In our research, we aimed to study the prevalence of MRI LMCE foci and their impact on neurodegeneration and disability in a cross-sectional cohort of patients with MS.

## 2. Methods

### 2.1. Study Population

54 MS patients included in the analysis are participants of a prospective study to determine the significance of LMCE in MS. The study was approved by the Local Ethics Committee of the FSBEI HE I.P. Pavlov SPbSMU. All subjects have signed the informed consent.

The inclusion criteria for this trial were as follows: (a) patients diagnosed with RRMS, SPMS, or PPMS according to McDonald 2005 or 2010 criteria, (b) age of 18–65 years, and (с) the ability to perform all study related procedures. Exclusion criteria were as follows: (a) previous or planned cytotoxic therapy (e.g., mitoxantrone), (b) previous or planned therapy with B-cell depleting agents (e.g., rituximab), (c) the presence of MS relapse and/or glucocorticosteroid treatment within 30 days preceding study entry, (d) pregnancy at the inclusion date, and (e) all contraindication for MRI (e.g., pacemakers, metal implants). All patients with RRMS and SPMS were on the 1st-line disease-modifying therapy (DMT), interferon-beta or glatiramer acetate, whereas the patients with PPMS did not receive DMT.

All subjects underwent physical and neurological examinations. EDSS score was calculated in all patients by a certified neurologist during their follow-up visits to the City MS Center every 3 months. To estimate the disease progression rate a Multiple Sclerosis Severity Score (MSSS) was calculated at the date of MRI according to the author's recommendations [13]. The integral number of relapses during the first year and first 5 years of the disease duration was calculated to measure the disease activity.

### 2.2. Laboratory Studies

Cerebrospinal fluid (CSF) samples were collected via atraumatic spinal tap and stored in the biobank of the City MS Center at −70°C immediately after collection. CSF tests were performed in those patients who consented to the procedure at the time of MS diagnosis confirmation. IgG-OCB status was assessed by isoelectric focusing and analyzed according to the international recommendations [14]. Other biomarkers of B-cell activation, kappa and lambda immunoglobulin free light chains (FLC), were measured using a novel ELISA assay (Polignost Ltd., St. Petersburg, Russia) based on monoclonal anti-k and anti-*λ* antibodies directed against cryptic epitopes of free FLC molecules.

### 2.3. MRI Acquisition Protocol

The protocol was adapted from the original study by Aloisi et al. Scans were acquired on General Electric (GE) Signa (General Electric Healthcare, Milwaukee, WI) 3T machine. MRI sequence protocol was as follows: manufacturer: General Electric; model discovery: 750 w; receive channels: 24; sequence name: CUBE; imaging plane: sagittal; imaging resolution (mm): 1 × 1 × 1; repetition time (TR, ms): 6500; echo time (TE, ms): 90; inversion time (TI, ms): 1956; flip angle (deg): 90; echo-train length: 140; bandwidth (Hz/pixel): 122; acquisition time (min:sec): 9:00.

A 3D T1 postcontrast sequence was performed immediately after intravenous infusion of a single dose of gadolinium 0.1 mmol/kg contrast agent, gadobutrol (Gadovist, Bayer AG, Leverkusen, Germany). 3D FLAIR sequence was acquired immediately after the end of T1 postcontrast acquisition. Precontrast 3D FLAIR scans were performed for all subjects.

Presence of LMCE was assessed by an experienced neuroradiologist (EM) with more than 10 years' experience in MS and a trained neuroscientist (GM) in the sphere of MS, masked to clinical and laboratory data, based on the interrater agreement. All discrepancies were processed by agreement. LMCE in the subarachnoid space was determined as definite when a signal intensity, greater than an intensity from an underlying brain parenchyma, was detected. All probable foci on postcontrast FLAIR were compared with precontrast FLAIR images to exclude a nonspecific increase of the signal. Only areas that were not detected on precontrast FLAIR were counted. T1 postcontrast enhancement of FLAIR post-Gd-enhancing foci was declared as normal and such foci were included in the analysis. All images were reviewed using OsirixViewer software (http://www.osirix-viewer.com) in the sagittal plane in original images and in coronal and axial views. Areas close to large blood vessels were carefully assessed. Possible foci, located close to large dural sinuses and cerebral veins, were analyzed with caution and indefinite foci were not included in the analysis.

Normalized whole-brain volume (NBV) and normalized white matter (NWMV) volumes were calculated using the SIENAX method of FLS package (https://fsl.fmrib.ox.ac.uk) [15]. A further segmentation analysis was performed with the FreeSurfer package (http://freesurfer.net) calculating the volumes of total grey matter, cortex, subcortical grey matter structures, ventricles, brainstem, and white matter hypointensities. To count the lesion volume, lesion masks were created for all patients using 3DSlicer (https://www.slicer.org) using T2 and FLAIR images as a source. All MRI analyses were performed by trained neuroscientists (GM) and underwent a quality control and were reviewed further by trained neuroradiologist (EM) at all stages of segmentation.

### 2.4. Statistical Analysis

The analysis was held in groups divided by the presence or absence of LMCE. All datasets were checked for normality with Kolmogorov-Smirnov's test. Comparison of discrete values was performed with Fisher's exact test. A comparison was performed with parametric (*t*-test) and nonparametric (Mann–Whitney *U* test) tests. All samples were checked for outliers using a standard procedure (ROUT test). Univariate analysis of covariance (ANCOVA), adjusted for age, sex, and disease duration, was performed to assess the effect of LMCE on brain atrophy estimates. Statistically significant difference was considered at *p* < 0.05. Data are presented as mean ± SEM or as median ± IQR based on the type of the distribution. All analyses were performed using GraphPad Prizm 7 and SPSS (Statistical Package for the Social Sciences).

## 3. Results

In total, 54 subjects were included in the study. All demographic characteristics had a nonnormal distribution. The median (IQR) age at the time of inclusion was 42 (22.5) years. According to the disease course, there were 36 (77%) patients with RRMS, 12 (22%) with SPMS, and 6 (11%) with PPMS. Male to female ratio was 1 : 1.7. Demographic data are presented in Table 1.

FLAIR postcontrast MRI was obtained in all included patients. LMCE was detected in 22 (41%) patients. Only scans with evident FLAIR postcontrast-enhancing foci, defined according to mentioned criteria, were included in the analysis. All equivocal areas were considered as LMCE-negative. Based on the LMCE status the patients were divided into LMCE-positive and LMCE-positive groups. Clinical, laboratory, and imaging data were obtained for these two groups. Results of calculations are presented in Table 2.

### 3.1. Demographic and Clinical Characteristics of the Subjects

Female patients prevailed in LMCE-positive group (73%) compared to LMCE-negative (54%), although the difference was not significant (*p* = 0.2615). Patients with detectable LMCE had a longer disease duration as compared to LMCE-negative patients (*p* = 0.0098) and were somewhat older although it was not significant (*p* = 0.071). No difference in the disease phenotype (RRMS versus progressive MS) was detected between groups (*p* = 0.148). LMCE-positive patients had a higher EDSS score, with median 4.0 versus 3.75 in LMCE-negative patients (*p* = 0.039). No significant difference in MS progression index MSSS was observed in two groups (6.12 versus 5.79, *p* = 0,864). Despite the higher EDSS score in LMCE-positive group, the analysis of relapse rate during the first year and 5 years from the disease onset revealed no differences between the groups (*p* = 0.2362 and *p* = 0.091, resp.).

The distribution of patients according to the disease duration revealed an increased prevalence of LMCE in patients with the disease duration over 20 years. The data are presented in Table 2. However, no difference was evident if the patients were ranged based on the age of MS clinical onset.

### 3.2. MRI Analysis

**Figure 1:**
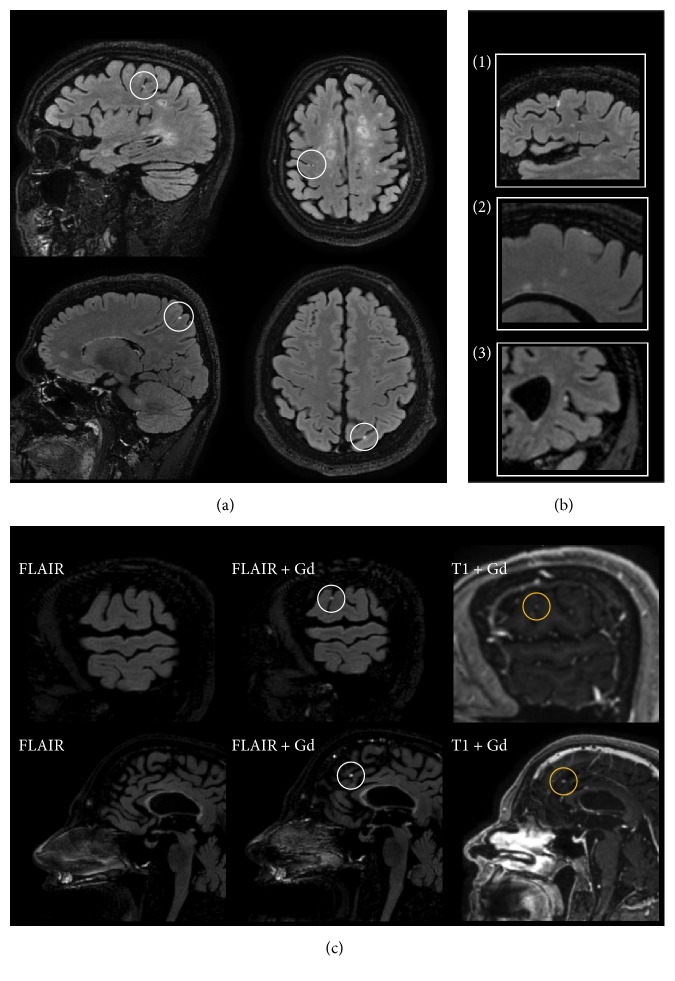
Main characteristics of LMCE. (a) Examples of localizations of LMCE on whole-brain images; (b) presentations of different types of LMCE foci: (1) linear, (2) plate-like, (3) nodular; (c) relationship between LMCE foci on precontrast FLAIR and postcontrast T1 and FLAIR.

In total, 54 LMCE foci were detected (Table 2). The number of foci in individual patients varied from 1 to 7. Regarding the shape, 31 (54%) foci had nodular, 17 (31%) plate-like, and 7 (13%) linear shape. 52 (96%) foci were localized in supratentorial regions and only 2 (4%) in infratentorial regions. Supratentorial foci were detected in both hemispheres: 32 (59%) in the left and 20 (41%) in the right. Predominant localization of the foci was in depths of sulci, 35 (65%), and only 19 (35%) were located superficially on the brain surface. In 35 (65%) FLAIR-contrast-enhancing leptomeningeal areas a weaker, but detectable, signal on T1 weighted postcontrast images was observed at the same place. The most eloquent LMCE foci and corresponding T1-enhancement areas are presented in Figure 1.

Both LMCE-positive and LMCE-negative groups revealed similar prevalence of T1 Gd-enhancing lesions according to Fisher's exact test. Mann–Whitney *U* test found no difference in the median T1 Gd-enhancing lesions count (*p* = 0,3842).

Unadjusted analysis of brain morphometry data was performed for all patients. MS patients with LMCE showed significantly smaller normalized brain volume (1389.8 versus 1426.2, *p* = 0,0462) and white matter volume (658.1 versus 678.6, *p* = 0,0468) compared to LMCE-negative patients. No differences in grey matter and cortical volumes were found between MS patients with and without LMCE (Table 3). A prominent ventricular enlargement was detected for LMCE-positive group (2.39 versus 1.73, *p* = 0.0168) together with the increased area of white matter hypointensities with a trend to significance (0.52 versus 0.35, *p* = 0.0509). Investigation of deep grey matter structures' volume, brainstem volume, revealed no significant difference in two groups (Table 3).

Analysis of data, adjusted for age, sex, and disease duration (ANCOVA), demonstrated a significant effect of LMCE on the cortex volume (*p* = 0.043, *F* = 2.529), total grey matter volume (*p* = 0.043, *F* = 2.54), and total ventricular volume (*p* = 0.039, *F* = 2.605) and a trend for a significant effect on thalamic volume (*p* = 0.051, *F* = 2.428) (Table 3). All other measurements found no significant effect of LMCE.

### 3.3. Laboratory Studies

Most patients in both groups were IgG-OCB-positive: LMCE-positive 88.9%; LMCE-negative 92.9% (*p* > 0.9999). Mean kappa-FLC concentration, although being higher in LMCE-positive group (LMCE-positive = 1.51 mcg/ml versus. LMCE-negative = 0.88 mcg/ml), did not reach statistical significance in comparison between groups with Mann–Whitney *U* test (*p* = 0.136), as did lambda-FLC (LMCE-negative = 0.45 mcg/ml versus. LMCE-positive = 0.5 mcg/ml, *p* = 0.4097).

## 4. Discussion

In this study, we assessed the frequency of leptomeningeal inflammation based on the detection of leptomeningeal contrast enhancement on the MRI FLAIR postcontrast imaging. Recent studies have demonstrated that LMCE was associated with significant cortical atrophy and cortical demyelination [9, 11]. These studies support findings of LMCE as a long-standing biomarker with a tendency to increase in progressive forms of MS. We showed in our study the LMCE frequency of 41%, which was higher than what had been published previously [9, 11]. This finding may be explained by rather high median EDSS score in the investigated cohort due to more patients on advanced stages of the disease, which may increase the proportion of LMCE-positive patients. In our study, LMCE was associated with older age, longer disease duration, and higher EDSS score. Despite a higher EDSS score, these patients did not experience more relapses than in LMCE-negative group. In the other studies, leptomeningeal inflammation was also associated with higher disease burden and progression rate [8]. This may indicate a more neurodegenerative nature of this disease phenomenon. This data is in line with the concept that LMCE is associated with more profound neurodegeneration [9].

Our study demonstrated that most LMCE foci were supratentorial and located in proximity to large meningeal vessels. No relation was found between LMCE and white matter lesions T1-contrast enhancement. It could be due to a distinct nature of these LMCE that may not be related to short-term bursts of inflammation in white matter.

Immunohistochemistry studies have demonstrated that ectopic perivascular lymphoid follicles have a strong B-cell component. In this study, we assessed some B-cell specific biomarkers. No difference was revealed for IgG-OCB positivity according to the LMCE status. These data are in line with the study by Absinta et al. [11]. B-cell activity can also be measured with immunoglobulin free light chains concentration [16]. In our study the concentrations of FLC kappa were higher for LMCE-positive patients although the difference was not significant. Studies of Ig-FLC concentrations with bigger sample size and other biomarkers of B-cell activity such as B-cell specific chemokines and activation factors may be helpful in identification of relation between LMCE and B-cell activity.

It was hypothesized that LMCE is associated with greater brain atrophy, especially with grey matter and cortical atrophy, based on the research by Zivadinov et al. [9]. Using the complex adjustment model, we detected the impact of LMCE on the total grey matter, cortical and thalamic atrophy, and ventricular enlargement in this cross-sectional study. Hence, LMCE-positive patients may be at risk of greater disability and faster disease progression in future. However, the rate of such progression remains to be evaluated in greater sample sizes and prospective studies. LMCE-positivity was not associated with greater relapse rate or T1 Gd-enhancement, so the effect on atrophy estimates is suggested to have another origin, probably neurodegenerative.

The origin of LMCE foci remains to be elusive. Still, little is known about the nature of these foci in MS. In the study by Absinta et al., it was shown that LMCE foci were associated with cell infiltrates around meningeal vessels. In our study, we could detect the colocalization of LMCE foci with T1-Gd-enhancement from meningeal vessel in 65% of cases, so, perhaps, it may reflect local disturbances of blood-meningeal barrier and CSF flow, for example, due to reactive fibrosis in the subarachnoid space.

The strength of the study was the 3D FLAIR MRI protocol with precontrast FLAIR acquisition that helped to exclude doubtful LMCE foci and a small slice thickness that helped to reveal even small areas of leptomeningeal enhancement.

## 5. Conclusion

LMCE is a feasible biomarker in multiple sclerosis with yet not fully determined significance. In our study, it was detected in 41% of patients and was associated with longer disease duration and a greater disability. LMCE was shown to be an independent and significant biomarker of grey matter, cortical and thalamic atrophy, and ventricular enlargement.

## Figures and Tables

**Table 1:** Cohort demographic characteristics.

Number of participants	54
*n* (%) female	34 (63%)
Median age at the time of MRI, years (IQR)	42 (22.5)
Median disease duration, months (IQR)	86 (149.05)
Median EDSS (25th, 75th percentile)	4 (2.5, 6.0)
Disease course at enrollment, *n* (%)	
(i) RRMS	36 (77%)
(ii) SPMS	12 (22%)
(iii) PPMS	6 (11%)

MRI: magnetic resonance imaging; EDSS: Expanded Disability Status Scale; MSSS: Multiple Sclerosis Severity Score; RRMS: relapsing-remitting multiple sclerosis, SPMS: secondary progressive multiple sclerosis, PPMS: primary progressive multiple sclerosis.

**Table 2:** Characteristics of leptomeningeal enhancement in the total cohort.

	LMCE-negative subgroup, *n* = 32 (59%)	LMCE-positive subgroup, *n* = 22 (41%)	*p* value
*n* (%) female	18 (56%)	16 (73%)	0.2615^*∗*^
Median age at MRI, years (IQR)	36.5 (24.25)	44.5 (22)	0.071
Median disease duration, months (IQR)	70.5 (123.05)	111 (156.25)	**0.0098**
MS phenotype, *n* (%)			
(i) Relapsing-remitting	24 (75%)	12 (25%)	
(ii) Progressive	8 (45.4%)	10 (54,6%)	0.148^*∗*^
Prevalence of LMCE according to disease duration:			
(i) 0–4 y	14 (74%)	5 (26%)	
(ii) 5–9 y	9 (60%)	6 (40%)	
(iii) 10–19 y	7 (54%)	6 (46%)	
(iv) ≥20 y	2 (29%)	5 (71%)	
Prevalence of LMCE according to age at onset			
(i) Before 19 y	5 (71%)	2 (29%)	
(ii) 20–29 y	13 (59%)	9 (41%)	
(iii) 30–39 y	6 (67%)	3 (33%)	
(iv) 40–49 y	5 (50%)	5 (50%)	
(v) ≥50 y	3 (50%)	3 (50%)	
Median EDSS (25th, 75th percentile)	3.75 (2.5, 4.5)	4 (3.25, 6.5)	**0.039**
Median MSSS (25th, 75th percentile)	5.79 (4.32, 7.08)	6.12 (3.62, 6.67)	0.864
Median number of relapses during first year (IQR)	2 (1)	1 (0.75)	0.2362
Median number of relapses during first 5 years (IQR)	2 (1)	2 (1)	0.091
Gd-enhancing T1 white matter lesions, *n* (%)			
(i) Present	7 (50%)	7 (50%)	
(ii) Absent	25 (62.5%)	15 (37.5%)	0.5306^*∗*^
Median number of T1 Gd-enhancing lesions (IQR)	0 (0)	0 (1)	0.3842
IgG-OCB positive^*∗∗*^, *n* (%)	24 (88.9%)	13 (92.9%)	>0.9999^*∗*^
Kappa-FLC concentration^*∗∗∗*^ in CSF, mcg/ml, mean ± SD	0.88 ± 0.94	1.51 ± 0.89	0.136
Lambda-FLC concentration^*∗∗*^ in CSF, mcg/ml, mean ± SD	0.45 ± 1.14	0.5 ± 0.6	0.4097

LMCE: leptomeningeal contrast enhancement; MRI: magnetic resonance imaging; EDSS: Expanded Disability Status Scale; MSSS: Multiple Sclerosis Severity Score; RRMS: relapsing-remitting multiple sclerosis; SPMS: secondary progressive multiple sclerosis; PPMS: primary progressive multiple sclerosis; OCB: IgG oligoclonal bands; FLC: immunoglobulin free light chains; CSF: cerebrospinal fluid; *∗*: to compare Fisher's exact test was used; *∗∗*: OCB LMCE-negative sample size = 27, OCB LMCE-positive sample size = 14; *∗∗∗*: kappa- and lambda-FLC concentrations: LM CE-negative sample size = 19, LM CE-positive sample size = 8. Data are presented as median with interquartile range (IQR) or as mean and standard deviation (SD) depending on the type of distribution. Significant differences are depicted in bold.

**Table 3:** Brain morphometry analysis according to the LMCE status.

	LMCE-negative group, *n* = 32 (59%)	LMCE-positive group, *n* = 22 (41%)	Unadjusted *p* value	Adjusted *p* value *Effect of LMCE*
NBV	1426.2 ± 47.8	1389.8 ± 82.9	**0.0462**	0.369
NTotalGMV	604.8 ± 63.9	582.4 ± 67.2	0.2117	**0.043**
NWMV	678.6 ± 29.6	658.1 ± 44.4	**0.0468**	0.180
NCV	452.1 ± 48.9	435.8 ± 53.2	0.2429	**0.043**
TotalVentV	23.2 ± 8.9	31.7 ± 16.8	**0.0168**	**0.039**
Thalamus	13.4 ± 1.6	12.8 ± 1.7	0.3277	*0.051*
Caudate	6.5 ± 1.5	6.6 ± 0.8	0.4656	0.363
Putamen	10.3 ± 1.5	9.5 ± 1.7	0.1059	0.229
Pallidum	2.7 ± 0.6	2.5 ± 0.4	0.3082	0.759
Hippocampus	8.1 ± 1.2	7.8 ± 1.3	0.4675	0.224
Brainstem	20.3 ± 2.4	19.6 ± 2.4	0.5965	0.40
WM hypointensities	4.6 ± 3.5	6.9 ± 4.9	0.0509	0.267
T2-LV	17.7 ± 3.8	18.4 ± 3.1	0.1236	0.254

LMCE: leptomeningeal contrast enhancement; NBV: normalized brain volume; NTotalGMV: normalized total grey matter volume; NWMV: normalized white matter volume; NCV: normalized cortical volume; TotalVentV: total ventricular volume (combined volumes of lateral, 3rd, and 4th ventricles); WM hypointensities: white matter hypointensities (“black holes”), T2-LV: volume of T2 lesions. Data for brain structures estimates are presented as mean ± SD in millilitres. Significant differences are depicted in bold.
